# “I’m Happy, Considering What I’ve Been Through”: An Interpretative Phenomenological Analysis of Quality of Life after Acute Brain Injury

**DOI:** 10.1007/s12028-026-02466-7

**Published:** 2026-03-03

**Authors:** Marie Dakeng, Pascal Antoine, Lionel Velly, Lydia Oujamaa, Jérôme Morel, Nory Elhadjene

**Affiliations:** 1 Department of Anesthesiology and Critical Care, Université Jean Monnet, CHU de Saint-Etienne, 42055 Saint-Étienne Cedex 2, France; 2 UMR 9193-SCALab - Sciences Cognitives Et Sciences Affectives, University Lille, CNRS, Lille, France; 3 Department of Anesthesiology and Critical Care Medicine, Aix Marseille University, University Hospital Timone, AP-HM, 13005 Marseille, France; 4 Aix Marseille University, CNRS, Inst Neurosci Timone, UMR7289, 13005 Marseille, France; 5 SRPR 42, Groupement de Coopération Sanitaire, Centre Hospitalier Universitaire de Saint-Étienne, Saint-Etienne, France; 6 GIN U1216, Grenoble Institute of Neurosciences, La Tronche, France; 7 Laboratory of Human Movement Biology, University Lyon, UJM-Saint-Etienne, Inter-University, EA 7424, 42023 Saint-Étienne, France

**Keywords:** Health-related quality of life, Acute brain injury, Withholding life-sustaining treatment, Neurocritical care unit, Ethics

## Abstract

**Background:**

In neurocritical care units (NCCU), decisions to withhold life-sustaining therapies are sometimes influenced by anticipated disability and quality of life (QoL) impairment, particularly when further interventions are deemed futile. However, health-related QoL (HRQoL) is inherently subjective and does not always correlate with disability levels. This qualitative, noninterventional study aimed to assess the relevance of HRQoL in ethical decision-making using interpretative phenomenological analysis (IPA) to investigate subjective HRQoL.

**Methods:**

Patients were interviewed by a single intensivist to assess their subjective QoL 2 years after their stay in an NCCU following an acute brain injury (ABI). The intensivist directed the interview toward HRQoL using a guide comprising limited and mostly open-ended questions. Audio recordings of the interviews were transcribed verbatim into Word narratives, analyzed in depth by two intensivists using IPA methodology and NVivo 14 software to enable exploration of patients’ lived experiences and personal QoL assessments.

**Results:**

A total of 14 patients were invited to the follow-up appointment, 7 for whom life-sustaining treatment had been withheld in the NCCU and 7 matched patients for whom this decision had not been made. Among the nine patients finally included, life-sustaining treatment had been withheld in four cases. Patients varied greatly in how they perceived and valued their QoL. While most valued relationships and independence, they expressed these values in different ways. Frustration with disability and support from relatives emerged as key motivators for rehabilitation. Despite their challenges, patients expressed gratitude for survival and pride in their progress and daily achievements. Overall, their experiences highlighted the deeply personal and subjective nature of disability and QoL assessment.

**Conclusions:**

HRQoL after ABI is highly subjective and should be considered with great caution in decisions to withhold life-sustaining treatment in NCCU. Further studies are warranted to improve outcome assessment after ABI and aid ethical decision-making.

**Supplementary Information:**

The online version contains supplementary material available at 10.1007/s12028-026-02466-7.

## Introduction

Acute brain injury (ABI), including traumatic brain injury, ischemic stroke, intracerebral hemorrhage, and subarachnoid hemorrhage, is a leading cause of disability worldwide and raises many ethical concerns in neurocritical care units (NCCU) [1, 2]. Beyond the variability of functional recovery, studies report a marked impairment in quality of life following stroke or traumatic brain injury (TBI) [3–7].

Quality of life (QoL) is a subjective measure defined by the World Health Organization as “an individual’s perception of their position in life in the context of the culture and value systems in which they live and in relation to their goals, expectations, standards, and concerns” [8]. QoL can only be accurately assessed by the individual [9], and there is no systematic correlation between disability level and quality of life, a phenomenon known as the disability paradox [10]. Health-related quality of life (HRQoL) has become a critical measure of care quality in recent decades, often assessed after ABI using tools such as the Short Form 36 (SF-36), Short Form 12 (SF-12), Quality of Life after Brain Injury (QOLIBRI), and QOLIBRI Overall Scale (QOLIBRI-OS) [11, 12]. HRQoL does not always correlate with neurological outcomes at the individual level [13]. After brain injury, HRQoL is negatively affected by factors such as length of hospital stay, duration of mechanical ventilation, and posttraumatic stress [14–16]. Results concerning the relationship between age or cognitive function and HRQoL are conflicting [5, 14, 15, 17]. Notably, resilience correlates with improved HRQoL [18, 19]. Anticipated poor neurological or QoL outcome may lead to decisions to withdraw or withhold life-sustaining therapies (WLST). These decisions concern up to 95% of patients with TBI or stroke who die in the intensive care unit (ICU) [4, 20–23].

Research on WLST shows significant associations with cultural and religious factors, resulting in considerable variation worldwide [20, 24–27]. Ethical dilemmas are particularly challenging in NCCU due to prognostic uncertainty and the unknown will of the patient [28]. They should be discussed carefully, as withholding life-sustaining therapies may create self-fulfilling prophecies that independently contribute to worse outcomes in patients with ABI [29, 30]. Decisions concerning WLST are often based on objective and known factors such as age, Glasgow Coma Scale (GCS) score, pupillary responses and comorbidities, as well as on expected neurological outcome and QoL [31, 32].

Expectations of HRQoL nevertheless remain subjective and uncertain at the time of ethical discussions. Qualitative methods are well suited to address this complex issue, requiring deep personal reflection and involving a limited number of individuals. Their use has progressively increased as a complement to quantitative methods. HRQoL after brain injury is particularly subjective, focusing on how individuals make sense of their experiences. This can be investigated using interpretative phenomenological analysis (IPA). First introduced by Jonathan Smith in 1996, IPA has since been described in more detail [33–35]. It is based on phenomenological, hermeneutic, and idiographic approaches, emphasizing the perception and interpretation of experiences, and analysis of individual cases. It represents a double hermeneutic, as the researcher interprets how people make sense of their experiences [35]. IPA is an inductive method, its results being derived from analyses of personal accounts rather than being based on pre-existing theories that could be confirmed or disproved [35]. Theme saturation is not sought, as IPA aims to capture the lived experiences of a small group rather than produce generalizable findings. It has already highlighted the grief that patients must process after a TBI or stroke to come to terms with significant changes and enable them to resume enjoyable activities, potentially return to work, and achieve social and professional reintegration [36, 37]. Rehabilitation and resilience in the face of disability appear to be key factors in patient recovery and reconstruction [38, 39]. IPA offers an appropriate methodological framework to explore patients’ lived experiences, an essential perspective in QoL evaluation. To the best of our knowledge, HRQoL after ABI has not been specifically explored using IPA.

The aim of this qualitative study was to explore the long-term HRQoL of patients previously admitted to a NCCU following an ABI, using IPA, a method emphasizing subjective experience, not requiring a specific sample size, typically involving small samples, and not necessitating theoretical saturation [35].

## Materials and Methods

Participants comprised patients admitted to a single NCCU for ABI in 2022, for whom withholding of life-sustaining therapies had been decided and who were still alive in 2024. WLST decisions were made during weekly ethical meetings within the department, attended by physicians, residents, and paramedical staff. A rehabilitation physician reviewed the cases beforehand to estimate the expected recovery, offering an external perspective. WLST refers to the non-initiation of specific life-sustaining therapies rather than the withdrawal of ongoing treatments. Decisions included no cardiopulmonary resuscitation (CPR), renal replacement therapy (RRT), high-dose vasopressors, prone positioning, or escalation to intensive neurocritical care. We matched them one to one with patients without WLST. Matching criteria included age, sex, initial GCS score, and type of injury (TBI, ischemic stroke, intracerebral hemorrhage, or subarachnoid hemorrhage). In total, 7 patients with WLST were included, for a total of 14 patients. All were invited to a post-NCCU follow-up appointment according to critical care routine. This follow-up was conducted by a single intensivist and served to assess the patient’s general health and current situation, including length of rehabilitation, date of return home, and any domestic support required. The intensivist then directed the interview toward HRQoL using a guide comprising limited and mostly open-ended questions (Supplementary File, Fig. 1). Participants were given a verbal explanation of the study and a detailed information sheet at the beginning of the consultation, permitting the entire session to be digitally audio-recorded.

**Fig. 1:**
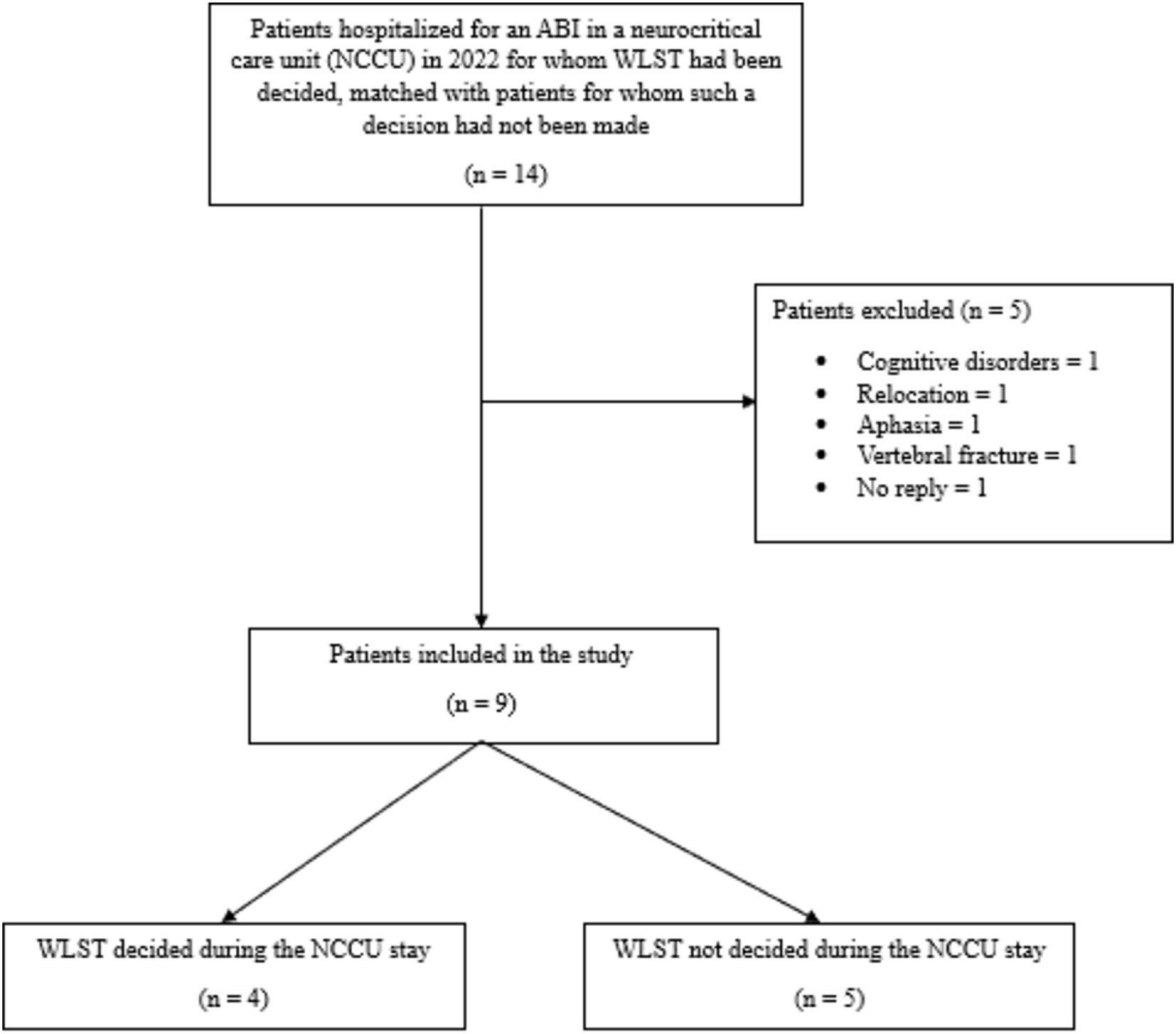
Flow-chart of the study

The audio recordings were transcribed verbatim into Word documents, analyzed by two intensivists using IPA methodology [35]. One intensivist analyzed the Word narrative directly while the other used NVivo 14 software. They first made exploratory notes, staying close to the patients’ accounts, but adding descriptive, linguistic, and conceptual notes to fully understand each patient’s perception of their experience. They then identified experiential statements describing personal psychological dynamics. These statements were subsequently grouped into themes, providing an overview of each patient’s main concerns and ways of making sense of them. Triangulation was conducted through investigator triangulation. Two intensivists (M.D. and N.E.) analyzed the transcripts independently, each unaware of the other’s interpretations. They then compared and discussed their findings to identify shared themes. Any divergent interpretations were discussed jointly. The individual experiential themes were then combined into group themes to provide a large overview including all patients. The most relevant group themes relating to patients’ perceptions of their HRQoL were selected for presentation here and were developed as narratives including verbatim extracts of patient responses. Only one disagreement occurred, which was resolved through discussion with the investigator who conducted the interview. A third researcher (P.A.) specialized in IPA re-read all the themes selected during transcript analysis and structured the results to provide a coherent overall framework highlighting individual perspectives. No feedback was provided to participants, although they were aware that the interviews were audio-recorded. In IPA, saturation is usually achieved once multiple themes emerge. In our study, 2 patients would have been sufficient, but we aimed to recruit 10–20 participants to ensure a range of perspectives. However, practical constraints limited further recruitment owing to the time-intensive nature of the IPA methodology.

Other data recorded included comorbidities and treatments, previous autonomy status, marital status, smoking, alcohol consumption, and medical and surgical interventions during NCCU stay, as well as ethical discussions and decisions, duration of mechanical ventilation, neurological status at NCCU discharge, and lengths of stay in the NCCU and medical ward. Glasgow Outcome Scale—Extended (GOSE), Clinical Frailty Scale (CFS), visual analog scale (VAS) for QoL, QOLIBRI-OS, SF-12, Functional Assessment of Chronic Illness Therapy—Fatigue (FACIT-F), instrumental activities of daily living (IADL) and Hospital Anxiety and Depression (HAD) scale scores were recorded during the consultation.

The GOSE is used worldwide to assess neurological outcome [11]. A score of 1 indicates death, a score of 2 denotes a vegetative state, and scores of 3–8 signify conditions ranging from severe disability to good recovery. A score of 4 or 5 and above usually indicates a favorable outcome.

Assessing QoL using a visual analog scale (VAS) is easy for patients and shows good correlation with other QoL scales [40]. The VAS used in our study ranged from 0 to 10, with 0 representing the worst perceived QoL and 10 denoting the best perceived QoL.

The QOLIBRI-OS is used to assess HRQoL in patients with TBI and stroke [41–43]. A score below 52 indicates low or impaired HRQoL [12].

The FACIT-F measures fatigue and has since been validated in many populations, including patients with stroke [44]. Scores range from 0 to 52, with higher scores indicating a better QoL. We used a score of 32 as a cut-off, as defined locally for standard care.

The IADL scale is based on eight items assessing the degree of dependency and has been validated in TBI and stroke populations [45, 46]. Scores range from 0 (low functioning, dependent) to 8 (high functioning, independent).

The HAD scale is used in populations manifesting psychological or somatic problems to assess symptoms of anxiety and depression. It attributes a score ranging from 0 to 21 for each of these symptoms [47]. Scores below 7 indicate no symptoms, from 8 to 10 uncertain symptoms, and above 11 definite symptoms. Patients with a score above 8 are advised to consult a psychologist.

The SF-12 has been validated worldwide to assess HRQoL and is widely used in patients with acute brain injury [48, 49]. It assesses both physical and mental health and provides a score between 0 and 100 for each of these aspects, respectively assessed on the physical component scale (PCS) and mental component scale (MCS). A score above 50 indicates a better-than-average HRQoL.

Use of these scales is part of standard care during follow-up.

The study protocol was approved by the local ethics committee (Comité d’éthique–Terre d’Ethique) on 4 June 4 2024 (IRBN672024/CHUSTE). According to French law, only verbal nonrefusal of the patient or his/her relative was required.

## Results

Among the 14 patients invited to the NCCU follow-up appointment, 9 agreed to participate, 5 being excluded for various reasons (Fig. 1). Patient characteristics at NCCU admission are presented in Table 1. The follow-up was conducted around 24 months after NCCU discharge. A total of five patients were accompanied to the follow-up appointment by relatives, who could help patients tell their stories and clarify their current situation. Patient characteristics at follow-up are presented in Table 2. A summary of each patient’s clinical history, along with the most relevant sections of their initial brain computed tomography (CT) scan, is available in the supplementary file.

**Table 1:** Baseline characteristics at neurocritical care unit admission

Patient	Sex	Age	CFS score	IADL score	Type of ABI	Initial GCS score	Decision to withhold life-sustaining treatment
1	Male	46	1	8	ICH	3	No
2	Female	76	1	8	SAH	11	Yes: no CPR, no RRT, no high-dose vasopressors
3	Female	74	1	8	SAH	12	No
4	Male	65	4	1	TBI	11	Yes: no CPR, no mechanical ventilation, no RRT, no high-dose vasopressors
5	Male	63	1	8	TBI	7	No
6	Male	60	2	8	TBI	11	Yes: no RRT, no CPR, no prone position
7	Female	68	7	2	TBI	10	Yes: no intensive neurocritical care
8	Female	72	1	8	TBI	11	No
9	Female	75	2	8	TBI	7	No

*ABI* acute brain injury, *CFS* Clinical Frailty Scale, *CPR* cardiopulmonary resuscitation, *GCS* Glasgow Coma Scale, *IADL* Instrumental Activities of Daily Living Scale, *ICH* intracerebral hemorrhage, *RRT* renal replacement therapy, *SAH* subarachnoid hemorrhage, *TBI* traumatic brain injury

**Table 2:** Patient characteristics at the neurocritical care unit follow-up appointment

Patient	Accompanied to the appointment by	GOSE score	IADL score	FACIT-F score	HAD scores (A; D)	SF-12 (PCS; MCS)	QOLIBRI-OS score	QoL (VAS) score	Patient’s quote concerning QoL
1	Mother	5	3	29	9; 6	45; 47	45.8	5	“I can already say that overall I’m fine.”
2	Husband	6	7	22	7; 10	34; 37	29	6	“Um, it’s a good quality of life, I think.”
3	Came alone	8	8	20	2; 9	33; 36	37.5	6	“That’s why my quality of life, for me, it’s… Lots of people would like to live like that”
4	Sister	6	1	35	5; 4	34; 40	37.5	6	*“I: Why do you say 6?* Patient 4: Well, if I say 1, that’s worth nothing, and if I say 10, that’s a lot…”
5	Wife	8	8	49	6; 3	50; 44	75	Between 5 and 10	*“I: So you think your quality of life is good overall?* Patient 5: Yes, it’s OK.”
6	Came alone	8	7	41	9; 4	39; 56	75	7	“I’m happy compared to where I’ve come from.”
7	Came alone	4	4	29	10; 5	44; 28	58	4	“I used to, well, live, but now … now I can’t do anything anymore.”
8	Husband	6	7	25	6; 8	33; 39	62.5	6	“I’m still living with my husband. That’s important, that’s why I got married 50 years ago”
9	Came alone	7	6	40	9; 11	56; 33	33.3	8	“It bothers me because I don’t feel like doing much at all. Life’s simply flat.”

*FACIT-F* Functional Assessment of Chronic Illness Therapy—Fatigue scale, *GOSE* Glasgow Outcome Scale—Extended, *HAD* Hospital Anxiety and Depression scale (A, anxiety, D, depression), *I* interviewer, *IADL* Instrumental Activities of Daily Living scale, *MCS* mental component score;, *PCS* physical component score, *QOL* quality of life, *QOLIBRI-OS* Quality of Life after Brain Injury—Overall Scale, *SF-12* Short Form 12, *VAS* visual analog scale

### Interpretative Phenomenologic Analysis

In-depth analysis of the patient narratives showed that most individuals experienced ABI as a unique life-challenging event. A specific characteristic was patient amnesia regarding the personal life event, often described as a black hole of varying length following ABI.

*Patient 1: It started strangely, I mean I had just left work, I had arrived home, I was going to…(...) And then nothing, nothing more (…) Apparently I fell. In fact, from the time I fell to the time I woke up, it was as if nothing had happened*

Despite what they had been through, patients were grateful for their current state thanks to their caregivers and relatives. Analysis of the interviews and questionnaires nevertheless highlighted a degree of ambivalence in how patients evaluated their quality of life. For instance, patient 1 reported: “I can already say that on the whole I’m fine,” while the quality-of-life questionnaires yielded scores that were borderline or below average [QOLIBRI-OS score of 45.8, SF-12 (PCS; MCS) scores of 45; 47, QoL VAS score of 5 out of 10]. In contrast, patient 9 stated that “Life’s simply flat.” This is consistent with her QOLIBRI-OS score of 33.3 and her SF-12 Mental Component Scale of 33, indicating an impaired quality of life. However, it contrasts with her self-rated quality-of-life VAS score of 8 out of 10 and her SF-12 physical component scale of 56, both suggesting a good quality of life. Overall, QoL assessment after ABI was a nuanced and multifaceted process, most often considered in relation to domains such as interpersonal relationships and autonomy.

**Fig. 2:**
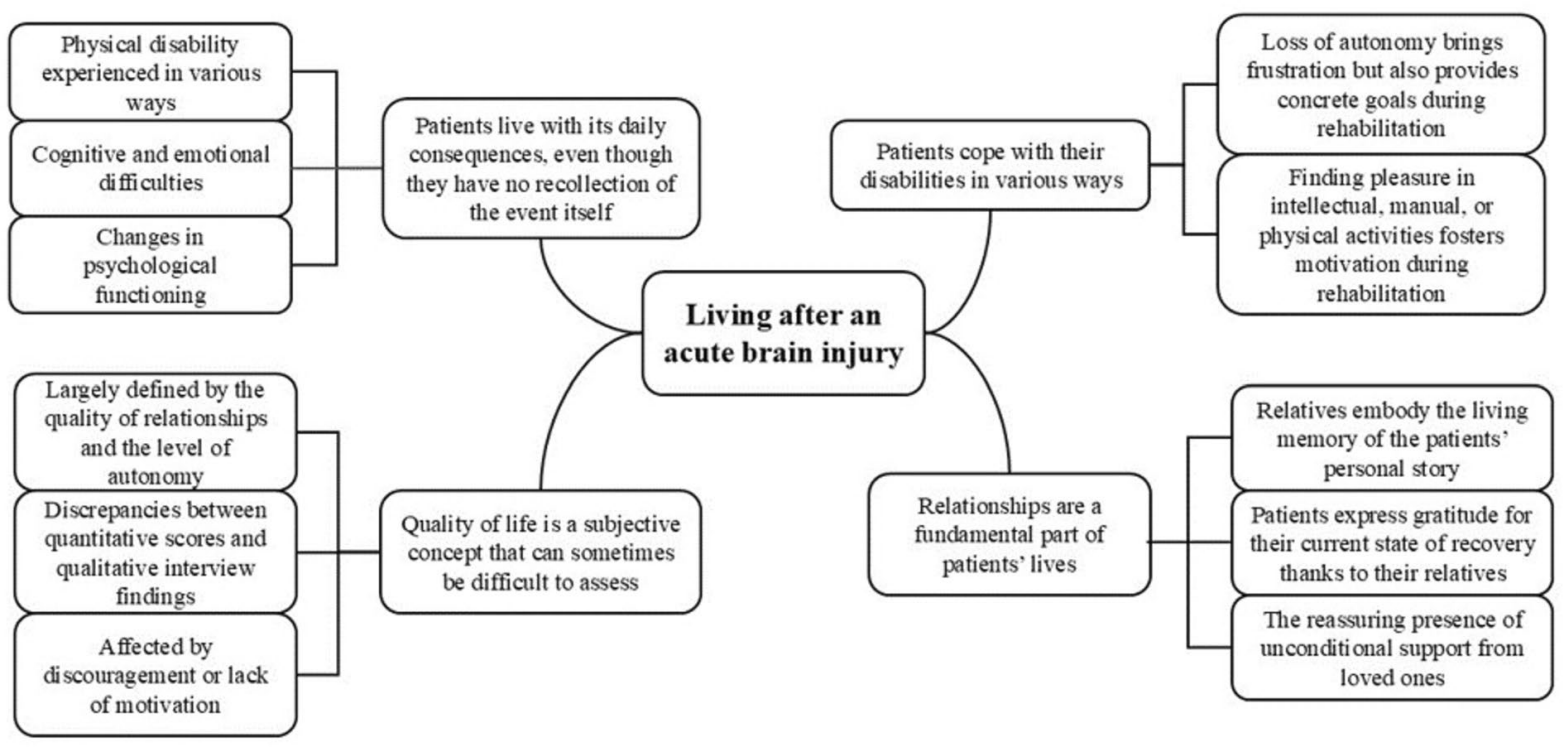
Patients’ perceptions of life after acute brain injury

The structure and narration of the results are based on the most relevant issues expressed by the patients, who mainly talked about the consequences of ABI on their daily lives, including their disabilities, interactions, and rehabilitation courses. ABI can lead to physical and intellectual disabilities, as well as psychological changes. These functional changes encouraged rehabilitation owing to the frustration of losing autonomy and the pleasure of participating in activities. Above all, close relationships helped patients to cope with their disabilities, improve their state and relate their stories. Figure 2 shows a general overview of these themes, which are subsequently examined in more detail.

### “I’m Not Completely the Same as I Was Before.”

Despite the amnesia surrounding ABI, patients experienced its everyday consequences physically, intellectually, and emotionally, both in their relationships and in society. Perception of their abilities and disabilities varied greatly between patients. Most patients were self-aware and gave objective descriptions of their disabilities and how they felt about these; however, two—patients 4 and 5—seemed to have poor insight, as they described their disabilities only vaguely, mentioning simply a strange feeling and rating their QoL on the visual analogue scale as if it were a theoretical question with a right or wrong answer. Patient 4 focused on the past during the interview, possibly a way of denying his disability.

*Patient 4: For a start, there’s nothing to do at home. And then, before, I did lots of things that I don’t do any more (…) I used to do my welding (…) Afterwards, no longer, I used to do things.*

Patients experienced physical and intellectual disabilities, such as loss of strength or speech problems, as well as psychological changes. Patients 1, 9, 6, and 7 exhibited anxiety-related symptomatology of uncertain significance, with HAD-A scores of 9 or 10. Upon closer examination, anxiety emerged as a particularly salient concern for the first two patients. Notably, although patient 9 demonstrated satisfactory physical recovery, she remained markedly impacted by psychological disturbances. She appreciated her daily routine, which made her feel safe, in contrast to the unknown. For patient 1, anxiety sometimes seemed to be blurred and he did not know exactly what he was afraid of. Both patients 1 and 9 were ambivalent and knew that fear hindered them in certain activities or relationships. Patient 9 referred to anxiety when she was alone but also when she met people. She recognized that the stress she experienced might be inappropriate for the situation.

*Patient 9: I’m very anxious (…) But it’s more fear of finding myself completely alone, stupid things (…) I didn’t know what they (her grandchildren) knew about my accident. I was a bit afraid of these contacts.*

Disability and psychological changes affected the patients’ position both within their family and in society. Patient 7, supported by her son, was worried about the negative impact of her disability on his life, such as not being able to have children. Patient 8 suffered from the increased vulnerability she experienced. She described in detail conflicts with her sister-in-law and her daughter-in-law, who regarded her as “crazy” because of her brain injury and its consequences.

*Patient 8: And since then, my relationship with my sister-in-law has been pretty tense (…) And she said at the end of the conversation “You’re crazy” And I couldn’t accept that. Just because I received a blow on my head, she thinks I live in a different world, that I don’t react normally, and so she said that I’m mad (…). And it’s my nature to brood on things that shock me. I have a job to stop thinking about them.*

On the positive side, patient 1 was now making an effort to talk to his daughters and was acting as a mediator in his extended family, whereas before the injury he described himself as angry.

*Patient 1: I used to be someone…I got very irritated about things. I didn’t always react appropriately…but now I’ve changed in lots of ways (…) I’m not the same person.*

Despite this positive change, patient 1 suffered greatly from increased vulnerability in his relationship with his ex-wife and from not being able to take care of his daughters as he used to. He tried to be a good father despite his disability. Returning to work could possibly harmonize his life as a source of pleasure, a way of caring for his daughters and a means of feeling valuable to society.

*Patient 1: First of all, I can’t work any longer; that’s for sure. I can’t drive any more, sorry, that’s finished (…) But the fact of having a job, I miss that enormously (…) I don’t want to be like someone who’s completely useless.*

Although functional recovery appeared broadly comparable, patients could differ in how they perceived their abilities across situations. This was the case for patients 2 and 8, both with a GOSE score of 6 (upper moderate disability) yet experiencing their driving limitations in different ways. Patient 2 accepted that she could no longer drive, whereas patient 8 was frustrated and felt trapped at home. She used the term “prisoner,” which was quite strong in relation to the situation and showed its importance for her.

*Patient 8: I’m not allowed to drive a car, so I’m a prisoner in my home. Just as in the hospital, I used to say that I was a prisoner in the hospital (…) My husband is retired, so he acts as my chauffeur, but I’ve lost my independence.*

### “I’m Delighted When I Know that I’ve been Able to…”

Discouragement in the face of disabilities and lack of desire experienced since the ABI were important issues, especially for patients 2, 8 and 9. Patient 9 explained that she was not interested in anything and that she had to force herself to be aware of her environment. Patient 2 highlighted the ambivalence between gratitude and discouragement. The latter was exacerbated by ageing and she related the difficulty of distinguishing between age-related decline and disability caused by her injury.

*Patient 2: So I want life to improve, but I know very well that won’t happen. After all, I’m old and so I know very well that life is not going to improve (…) But of course, I know I should be grateful and I am. But all the same, from time to time I don’t feel like doing anything, I mean I’ve had enough. There are some days when I just want to sleep all the time. In the morning I want to go on sleeping. But I have to get up, I have no choice, because they (the nurses) are going to arrive, so that’s it. And anyway, I know very well that I can’t just go on sleeping. That’s impossible. But there are days when I feel really discouraged. That’s a fact. But as for consulting a psychologist, sorry, but that doesn’t appeal to me.*

Being aware of one’s disability is also an essential intellectual step toward rehabilitation. Disability led to loss of autonomy, which was a source of frustration, independently of IADL score. Patient 4, whose IADL score was 1 (dependent), did not appear concerned about his dependence, whereas patient 3, with a score of 8 (independent), placed considerable emphasis on her efforts to maintain autonomy. She described her resumption of many tasks and emphasized the routine she had achieved. She could not bear to be cared for by someone else. Use of the terms “never” and “always” highlighted the consistency of her autonomous status. She refused to complain, reflecting her will to cope with her disability by herself.

*Patient 3: Nobody has ever injected my insulin. I’ve always done it myself (…) My children have never taken care of anything. People are amazed. First of all, because I don’t complain. I do whatever needs doing.*

Frustration appeared therefore as a key motivator for rehabilitation yet it allowed patients to set concrete goals and to see progress. This involves achieving small improvements over a long period of time and is supported by a daily routine. Patient 1 described the housework he could do, as well as writing in his diary.

*Patient 1: I write that now, I never did so before, but now I write something every day (…) At first, it was difficult, but little by little (…) I’m starting to re-read what I’ve written here (…) As for doing the cooking, I do it, yes, with someone else. When someone is preparing a meal, I help in various ways (…) She shows me lots of things.*

Many patients insisted on returning home after their hospital and rehabilitation stay. Remembering the exact date of this event showed how important this step was for patients and their family, like an anniversary. Only patient 1 had not yet returned home and expressed difficulties in handling this situation.

*Patient 1: It’s lucky my mother’s there because by myself I couldn’t… (…) But all the same, I would like to be by myself a bit at home, so as not to bother her too much, but I wouldn’t want her to be too far away either, as I don’t want to be worried…*

Enjoying physical, intellectual, or manual activities was a source of motivation and helped rehabilitation. Patient 8 emphasized manual activities, which allowed her to recover her dexterity and maintain a social life. It was also a step toward taking up painting on china again, an activity she immensely enjoyed and that therefore represented an important source of motivation.

*Patient 8: I achieved my rehabilitation by myself, with my hands. I have taken up patchworking and sewing by hand again (…) Because my absolutely favorite activity is painting on china. That’s breakable. If a piece of fabric falls, it doesn’t break, but china…*

Some patients reported that feeling and seeking security is important for rehabilitation. Patient 5 felt safer riding a bike alone than joining a team, as it allowed him to go at his own pace without pressure. He also attached importance to tasks he had always done and had now started doing again.

### “People. Well Yes, I Think that’s the Most Important Thing, Really.”

Relationships are the living memory of the patients’ own story. Relatives remembered the patient’s ABI and NCCU stay, unlike the patients themselves. This enabled them to recount details of the patient’s experience and to clarify events when the patient asked questions. Patients were generally interested in hearing details of their own hospital course and seemed quite comfortable with this part of their own story, in contrast to the relatives, for whom this period had been a traumatic experience.

*Patient 6: “When my family explained what had happened to me, well, I was happy about that.”*

Close relatives encouraged patients to exercise caution when carrying out certain activities, owing to the trauma they had experienced because of the ABI. Patient 6 said that his family was more worried about him than he was himself because they remembered his accident.

*Patient 6: I take out the tractor, and all that, use the chain saw (…) I’m not afraid to use the chain saw, I just use it (…) It’s a pleasure for me (…) It’s important. I’m happy when I think about it. I don’t know, I’m happy considering what I’ve been through (…) Often, my son says « Dad, what you’ve been through… Stay calm » And my wife, even better. She was the one who found me…*

Many patients placed great value on their relationships and measured their QoL by these. Patient 2 regarded her relationship with her husband as the cornerstone of her life and stressed the importance of her relationships with her children and friends.

*Patient 2: It’s my husband. He’s a dear. Because without him, I don’t know what would have happened to me. I owe a lot to the doctors and the nurses, but also to him. He’s always been by my side and… (…) What helps me to do well? My husband, for a start. Our children. It’s true. Because they’ve been wonderful. And also some friends of course.*

Owing to their disability, patients rely on their relatives. Specifically, patient 1 and 2 recognized the unwavering support of their relatives in the rehabilitation context. Patient 1 was grateful to his mother but knew that regaining autonomy would be a way of relieving her. He also emphasized the importance of the support he received from his best friend, expressing the value of this support in several rephrased statements.

*Patient 1: What’s good is that there are still some people who’ve given me tremendous support, including my best friend, who has been… He’s always been there when I needed him (…) and my mother as well, who has supported me enormously. That’s enormous.*

### WLST and IPA Outcomes

Of the four patients for whom life-sustaining therapies were withheld, patient 2 received no CPR, RRT, or high-dose vasopressors; patient 4 received no CPR, mechanical ventilation, or RRT; patient 6 received no RRT, CPR, or prone positioning; and patient 7 received no intensive neurocritical care. Functional outcomes in this group varied, with GOSE scores from 4 to 6 and IADL scores from 2 to 7. IPA narratives revealed a complex mixture of gratitude, anxiety, and ambivalence, illustrating that subjective quality of life did not strictly reflect the clinical limitations initially imposed.

The five patients without WLST (patients 1, 3, 5, 8, and 9) experienced no restrictions in life-sustaining therapies, with GOSE scores ranging from 5 to 8 and IADL scores from 3 to 8. Their IPA narratives highlighted a broad spectrum of experiences, from satisfaction and engagement in daily life to frustration and discouragement, demonstrating that full neurocritical care does not guarantee a uniform perception of recovery.

## Discussion

This study used IPA to explore HRQoL in patients admitted to an NCCU following ABI. In-depth analysis of patients’ narratives highlighted the subjective nature of perceived disability and HRQoL. Relationships and autonomy emerged as key themes, rehabilitation being driven by family support and desire for independence. Some patients struggled to assess their HRQoL, expressing ambivalence.

Qualitative studies of HRQoL after ABI showed similar challenges. Accepting not being the same person as before was part of a new reality, characterized by physical, psychological, or cognitive changes. This significant issue for patients often leads to frustration or inner conflict [19, 37, 50, 51]. Facing disability can cause fear but also pride through overcoming obstacles [52]. Patients recognize the value of support from relatives or external help in coping with their situation [19, 50]. Some studies also highlight the struggle to find meaning, fears about the future, and awareness of core values, [19, 50], themes not explored in our study through specific questions (Supplementary File, Fig. 1). However, acceptance of their situation allows patients to experience a relatively good HRQoL [51]. The TBI resilience model suggests that adversity requires adaptation in emotional, cognitive, and behavioral domains [53]. Emotional functioning and self-efficacy are therefore positively correlated with QOL, whereas self-awareness and cognitive functioning are negatively correlated [17], highlighting the complexity of human psychology. Improvement of personal factors during rehabilitation may enhance HRQoL [17], indicating the importance of rehabilitation after ABI. Our patients valued their rehabilitation sessions, despite some shortcomings, and were delighted with their achievements. The positive impact of exercise and combined cognitive and psychological interventions on QoL is well documented [54, 55]. To be effective, rehabilitation should be tailored to each patient’s needs. The International Classification of Functioning (ICF) distinguishes between “impairment,” “activity limitation,” and “participation restriction,” all of which fall under “disability” [56]. Similar impairments may therefore have different consequences depending on personal activities and the environment, as expressed by our patients. Beyond physical abilities, participants considered the people around them to be the most important factor. Patients with ABI want to play a role in their family, maintain friendships, and be socially integrated [57]. Seven of the nine patients presented anxiety or depressive symptoms, as assessed by the HAD scale, but only two accepted an appointment with a psychologist, possibly owing to negative perceptions of the usefulness of psychologists and societal attitudes toward mental health care.

Participants expressed personal changes, the value they placed on small achievements, and the support they received from relatives in different ways, but none of these factors was predictable. Furthermore, participants rated their QoL differently. The unpredictability of people’s reactions to disability and the subjective nature of QoL explain the difficulty of predict HRQoL. In fact, predicting patients’ QoL is much more difficult than predicting their neurological outcome [58]. This lack of accuracy reduces the relevance of HRQoL to ethical NCCU discussions. As patient-reported outcomes gain importance, the GOSE score becomes increasingly less appropriate for assessing recovery. A core outcome set is needed to evaluate ABI recovery from both patient and physician perspectives [59]. At present, neurological outcomes should be considered cautiously in ethical discussions, a recent study showing that NCCU variables mainly correlate with mortality [60]. Advanced directives and surrogate reports may be biased, failing to capture patients’ disability and resilience [9, 53, 61]. ABI often occurs in healthy people who never expected such an event or wanted to be dependent. All our patients expressed gratitude for the care they received and their luck in surviving. Given the complexity of predicting and assessing ABI outcomes, neuro-intensivists should be cautious about withholding life-sustaining treatment on this basis.

Our study has several limitations. First, it focused solely on nine patients admitted to a NCCU after ABI. It is difficult to extrapolate our findings to all patients with ABI, and even more so to other critically ill patients, as brain injury tends to be more disabling than other causes of intensive care unit (ICU) admission, such as sepsis or acute respiratory distress syndrome (ARDS). Furthermore, it is likely that other people would describe their experience of an acute brain injury differently. We do not claim to have explored the full range of possible experiences. In this study, we only included patients for whom life-sustaining therapies had been withheld. Patients who underwent withdrawal of ongoing treatments died during their stay in the NCCU and could not be included in the study. This selection criterion may limit the generalizability of our findings to patients facing end-of-life decisions in neurocritical care more broadly. Second, the analysis was performed only once, at 2 years after NCCU discharge, precluding assessment of the QoL trajectory or disability over time and not accounting for potential changes in QoL determinants. However, at 6 months after ABI, patients often still manifest significant disability, making qualitative analysis difficult. Long-term QoL analysis is in fact a strength of our study. Third, our study excluded patients for whom a semi-structured interview at the hospital was not feasible (owing to cognitive impairment, aphasia, relocation, or vertebral fracture), introducing a bias. Assessing QoL in this group of patients would have required a different methodological approach or additional resources (e.g., home visits by the researcher). This study underscores the importance of longer follow-up for NCCU patients with ABI and the development of accessible alternatives for those unable to attend in-person visits.

Moreover, the interviews were conducted during a physical NCCU follow-up. The time spent with the patient was therefore not devoted exclusively to exploring QoL but also included medical assessment. An asymmetrical relationship between the researcher and the patient may inadvertently have been established, which could have unconsciously influenced the interview. However, the face-to-face interviews fostered trust and encouraged patients to discuss topics they might not have raised otherwise. Questions about weight gain, physical capacity, and domestic help prompted reflections on body image, strength recovery, and coping with everyday life limitations.

Although the presence of relatives may have limited patients’ openness, it was generally helpful. Relatives provided important information about the patient’s recovery and daily life, especially as many patients manifested amnesia or speech difficulties hampering communication. Relatives play an important role throughout the hospital and rehabilitation process and their support should not be underestimated [62].

In addition, we focused on patients’ personal experience after ABI, with or without the withholding of life-sustaining treatment in the NCCU. A key question in this context is the intensity of NCCU treatment and how patients experience this aggressive care. However, all patients reported partial or complete amnesia, which limited the interviewer’s ability to explore their perceived NCCU experience. Only two patients remembered parts of their ICU stay and expressed gratitude for the care they received, while the others, despite remembering nothing, were nevertheless grateful for this care.

Finally, we used IPA for in-depth qualitative analysis, as opposed to quantitative methods. This highlighted the psychological complexity of patient perceptions of HRQoL and the need to address this issue from the patient’s perspective.

## Conclusions

QoL after ABI is complex and influenced by many personal factors. It is affected more by participation restrictions than by objective impairment severity and can be significantly improved by tailored care and rehabilitation after NCCU discharge. HRQoL after ABI is therefore highly subjective and should be considered with great caution in decisions on withholding life-sustaining treatment in NCCU. Further studies are warranted to improve outcome assessment after ABI and aid ethical decision-making by neurocritical care physicians. IPA offers a valuable approach through its personalized and in-depth exploration of individual lived experiences.

## Supplementary Information

Below is the link to the electronic supplementary material.

Supplementary file1 (DOCX 619 kb)

## Data Availability

The datasets used and/or analyzed during the current study are available from the corresponding author on reasonable request.

## Abbreviations

ABI: Acute brain injury
ARDS: Acute respiratory distress syndrome
CFS: Clinical frailty scale
FACIT-F: Functional assessment of chronic illness therapy—fatigue
GCS: Glasgow coma scale
GOSE: Glasgow outcome scale—extended
HAD: Hospital Anxiety and Depression
HRQOL: Health-related quality of life
IADL: Instrumental activities of daily living
ICF: International classification of functioning
ICU: Intensive care unit
IPA: Interpretative phenomenological analysis
NCCU: Neurocritical care unit
QoL: Quality of life
QOLIBRI: Quality of life after brain injury
QOLIBRI-OS: Quality of Life after Brain Injury—Overall Scale
SF-12: Short Form 12
TBI: Traumatic brain injury
VAS: Visual analog scale
WLST: Withdrawal or withholding of life-sustaining therapies
