# Supplementary material for: “I’m Happy, Considering What I’ve Been Through”: An Interpretative Phenomenological Analysis of Quality of Life after Acute Brain Injury
Source: Neurocrit Care. 2026 Mar 3;45(1):260–72. doi: 10.1007/s12028-026-02466-7 (PMC13369703; doi:10.1007/s12028-026-02466-7)
Supplement: Supplementary file 1 — Supplementary file1 (DOCX 619 kb) [file 12028_2026_2466_MOESM1_ESM.docx]

**Supplementary File**

1. Can you tell me what you have understood about your acute brain injury?
2. Can you tell me what has happened to you since you were admitted to hospital with an acute brain injury?
3. What do you think about the treatments you received during your stay in the neurological intensive care unit?
4. Have you experienced any physical pain, difficulty sleeping, memory problems or any other problems since your acute brain injury?
5. What changes have you noticed in your life since you were admitted to hospital with an acute brain injury?
6. On a scale of 0 to 10, how do you rate your quality of life? 0 being the worst and 10 being the best you can imagine.
7. What criteria do you use to rate your quality of life?
8. What are the most valuable things in your current life?

**Figure 1.** Interview guide.

**Patients’ clinical history**

**Case #1**

A 46-year-old man, delivery driver, divorced with two children, living alone in an apartment (Instrumental Activities of Daily Living (IADL) 8/8, Clincal frailty scale (CFS) 2/9), was admitted to the neurocritical care unit with a Glasgow Coma Scale (GCS) score of 3 and reactive pinpoint pupils. The initial CT scan revealed a temporo-parieto-occipital hemorrhagic intracerebral hematoma. He underwent hematoma evacuation with bone flap replacement, and the initial intracranial pressure (ICP) was 8 mmHg. On day 1, he developed refractory intracranial hypertension under thiopental, requiring decompressive craniectomy. Brain MRI performed on day 2 showed embolic-appearing ischemic lesions and several lacunar infarcts. Up to day 22, his course was complicated by acute respiratory distress syndrome (ARDS) requiring mechanical ventilation, which delayed sedation weaning. By day 28, neurological improvement was noted, and he was extubated. On day 36, his GCS was 14 (E4V4M6) with confusion, right homonymous hemianopia, and visual agnosia, and he was transferred to the post-ICU rehabilitation unit.

At month 2, he was discharged from the post-ICU rehabilitation unit and transferred to a rehabilitation facility. At month 5, he was discharged home to his mother’s residence, as he could not return to his own apartment due to architectural barriers (no elevator, bathtub) and distance from outpatient rehabilitation services.

At the 21-month post-ICU follow-up, the patient still lived with his mother and continued intensive outpatient rehabilitation. He remained highly dependent (IADL 3/8, CFS 5/9) and reported anxiety when alone. Physical recovery was satisfactory, and he had resumed reading and writing. However, due to persistent visual deficits, he was unable to return to his occupation as a delivery driver and was considering vocational retraining with recognition as a disabled worker.


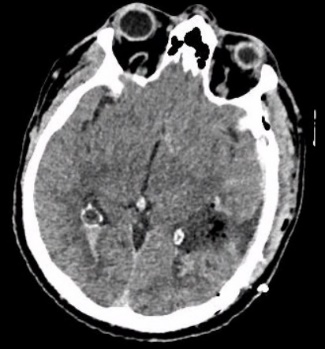


**Figure 2.** Brain CT scan of case #1 at H+24 after admission in ICU and after neurosurgery.

**Case #2**

A 75-year-old woman, married with three children and previously independent at home (IADL 8/8, CFS 2/9), was admitted with a subarachnoid hemorrhage (SAH) Fisher grade IV and WFNS (World Federation of Neurosurgical Societies) grade 5, associated with a left temporal hemorrhagic intracerebral hematoma secondary to rupture of a left middle cerebral artery aneurysm.

On day 0, she developed refractory intracranial hypertension requiring insertion of an external ventricular drain (EVD). The aneurysm was secured on day 3 with a Flow Diverter stent. On day 8, she experienced a right sylvian vasospasm treated with intravenous and in-situ milrinone, followed by ventilator-associated pneumonia on day 10. On day 20, her neurological status remained poor with a GCS of 6 (E1V1M4). Following an ethical discussion, the team decided not to perform cardiopulmonary resuscitation (CPR), renal replacement therapy (RRT), or high-dose vasopressor administration.

By day 35, her condition improved to a GCS of 12 (E4V2M6) after six days of extubation, allowing transfer to the post-ICU rehabilitation unit. At month 2, she underwent ventriculoperitoneal shunt placement for chronic hydrocephalus, which led to marked neurological improvement. She was discharged from the post-ICU rehabilitation unit at month 4 and transferred to a rehabilitation facility, then returned home by month 8.

At the 20-month post-ICU follow-up, the patient reported residual fatigue and decreased muscle strength (Hand Grip Test: 13 kg), along with functional limitations due to digital ankylosis. She had regained a good level of independence, requiring assistance only for bathing and home help twice a week (IADL 7/8, CFS 2/9). She had resumed walking, although ambulation was limited by probable peripheral arterial disease, which was under investigation.

**
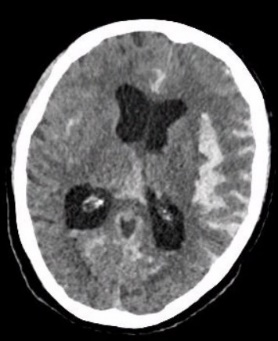

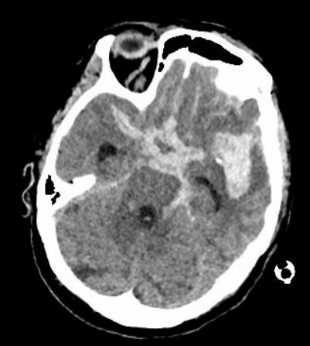
**

**Figure 3.** Brain CT scan of case #2 at ICU admission.

**Case #3**

A 74-year-old woman, living alone with two children and previously independent at home (IADL 8/8, CFS 2/9), was admitted with a SAH Fisher grade IV and WFNS grade 1, associated with intraventricular hemorrhage due to rupture of a posterior inferior cerebellar artery (PICA) aneurysm.

On day 1, the aneurysm was secured using a Flow Diverter stent. On day 10, intravenous milrinone therapy was initiated for a left sylvian vasospasm. The patient was extubated on day 11 and had a GCS score of 15 by day 17, allowing transfer to the neurosurgery ward.

At month 3, she returned home.

At the 20-month post-ICU follow-up, the patient was living alone, supported by her neighbors and maintaining close contact with her two sons. She required minimal home assistance, including nursing visits for medication management and domestic help twice weekly. Physical and cognitive recovery were satisfactory (IADL 8/8, CFS 2/9). However, she presented with significant apathy, describing the need to make an effort to initiate daily activities.

**
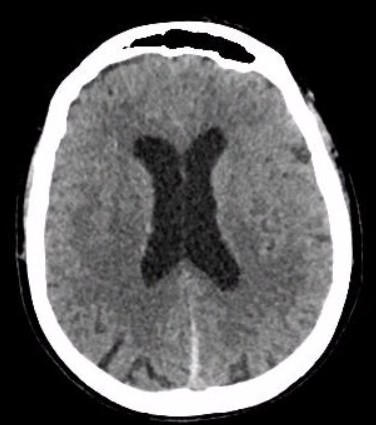

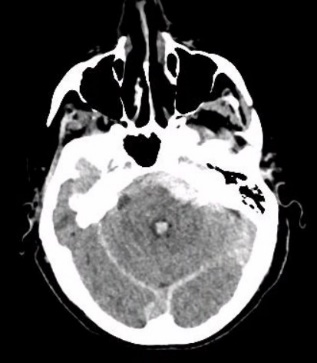

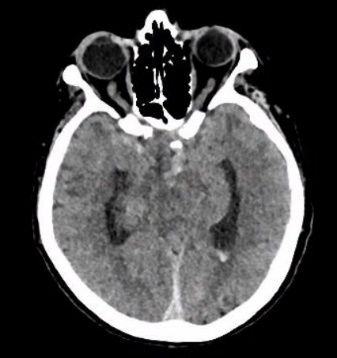
**

**Figure 4.** Brain CT scan of case #3 at ICU admission.

**Case #4**

A 64-year-old man with a history of dilated cardiomyopathy (LVEF 48%) and chronic alcoholism under legal guardianship, living alone (IADL 1/8, CFS 4/9), was admitted following a mild traumatic brain injury (GCS 14) with bifrontal hemorrhagic contusions, subarachnoid hemorrhage, and subdural hematoma without CT evidence of raised intracranial pressure. He was initially admitted to the step-down unit, then transferred to the intensive care unit (ICU) due to progression of bitemporal contusions on CT, agitation related to alcohol withdrawal, and septic shock secondary to aspiration pneumonia.

By day 4, both neurological and respiratory improvements were observed. During an ethical discussion, the medical team decided on limitations of therapy: no CPR, no mechanical ventilation, no RRT, and no high-dose vasopressors. On day 9, the patient was calm and cooperative, with a GCS of 14 (E4V4M6) and marked confusion, allowing transfer back to the step-down unit.

At month 2, he was transferred to a rehabilitation facility, then to a nursing home at month 4, and returned to his home at month 7.

At the 24-month post-ICU follow-up, the patient was living alone at home with extensive external support, including nurse visits, home assistance, and meal delivery. He had regained good physical condition but continued to report significant memory difficulties. His functional scores remained stable (IADL 1/8, CFS 4/9).

**
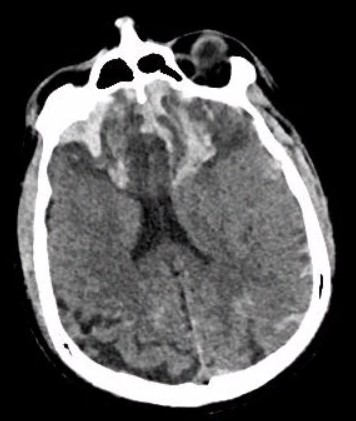
** **
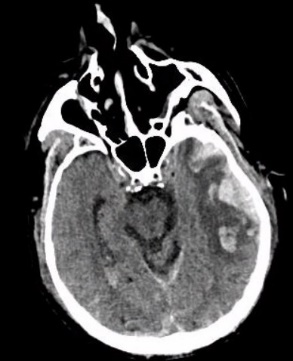
**

**Figure 5.** Brain CT scan of case #4 at ICU admission.

**Case #5**

A 63-year-old man, married and living independently at home (IADL 8/8, CFS 1/9), was admitted following an initial mild traumatic brain injury (GCS 15) with secondary neurological deterioration to a GCS of 7. The initial CT scan revealed a traumatic SAH and a left frontoparietal subdural hematoma without mass effect or ventricular dilation.

He was admitted to the neuro–intensive care unit on day 0. Sedation was discontinued on day 3, and the patient was extubated. By day 11, he had regained full consciousness with a GCS of 15 and was subsequently transferred to the general medicine ward.

At month 2, he returned home following a period of rehabilitation.

At the 25-month post-ICU follow-up, the patient demonstrated excellent physical and cognitive recovery and had resumed all previous activities, including cycling and do-it-yourself projects. He required no home assistance, with functional scores remaining optimal (IADL 8/8, CFS 1/9).

**
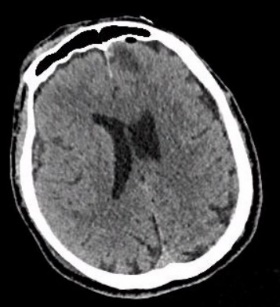
** **
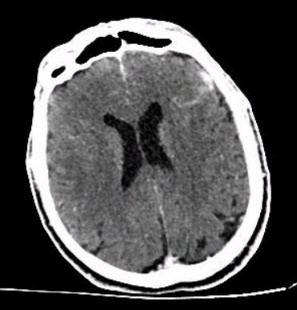
**

**Figure 6.** Brain CT scan of case #5 at ICU admission.

**Case #6**

A 60-year-old man with a history of severe ischemic heart disease (LVEF 29%, grade 3 mitral regurgitation, implantable cardioverter-defibrillator), squamous cell carcinoma of the piriform sinus treated with chemoradiotherapy and cervical surgery, moderate chronic obstructive pulmonary disease (COPD), and chronic alcohol use, was admitted following a mild traumatic brain injury. He was married, had two children, and lived independently at home (IADL 8/8, CFS 2/9).

The initial neurological assessment showed a GCS of 15, followed by secondary deterioration to a GCS of 10. The CT scan revealed diffuse subarachnoid hemorrhage, a left parieto-occipital subdural hematoma, and a left temporal hematoma. On day 0, the patient developed refractory intracranial hypertension requiring evacuation of the left temporal hematoma and decompressive craniectomy. On day 2, he experienced severe cardiogenic shock. Following an ethical discussion, therapeutic limitations were set: no RRT, no CPR, and no prone positioning.

By day 10, cardiac function had improved. The neurological examination showed a GCS of 10 (E3V1M6), and an external lumbar drain was placed due to external hydrocephalus. On day 17, extubation failed because of ICU-acquired neuromyopathy, but a second extubation on day 25 was successful. On day 33, a gastrostomy tube was inserted, and cardiac function had returned to baseline. At that time, the patient had a GCS of 14 (E4V4M6), with fluctuating temporal and spatial disorientation, occasional speech difficulties, and preserved mobility of all four limbs, though he was unable to stand. He was transferred to the step-down unit on day 38.

At month 1, the patient was admitted to the post-ICU rehabilitation unit, followed by transfer to a rehabilitation facility at month 2. Cranioplasty was performed at month 10, and he returned home at month 11.

At the 25-month post-ICU follow-up, the patient exhibited good physical recovery and autonomy, having resumed all his usual activities, including gardening and do-it-yourself projects (IADL 7/8, CFS 2/9). Cognitively, he reported persistent memory issues and occasional word-finding difficulties. He continues regular cardiology follow-up, and a heart transplant is currently being considered.


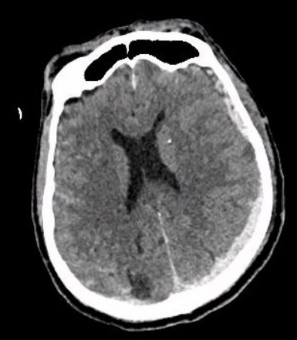

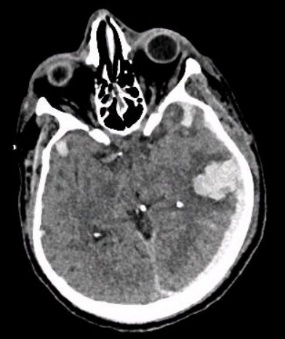


**Figure 7.** Brain CT scan of case #6 at ICU admission.

**Case #7**

A 68-year-old woman with a medical history of progressive multiple sclerosis, systemic lupus erythematosus with antiphospholipid syndrome and prior catastrophic manifestations including multiple strokes, infective endocarditis with cerebral septic emboli, and severe colitis requiring colostomy, was admitted following a mild traumatic brain injury. She lived alone and had two children, receiving substantial home support including twice-daily nursing visits and caregiver assistance for personal hygiene, meals, and household tasks (IADL 6/8, CFS 7/9).

Initial CT imaging revealed a left-sided subdural hematoma. On day 2, she experienced neurological deterioration with a GCS of 10 (E4V1M5), secondary to hydrocephalus and the development of a right frontal intraparenchymal hematoma. An ethical discussion led to the decision to limit neurocritical interventions. An EVD was placed under neuro-sedation and local anesthesia. By day 15, the EVD was removed, and her GCS had improved to 15, allowing transfer to the neurosurgery ward.

At month 2, she was discharged from hospitalization and transferred to a rehabilitation facility, and by month 3, she returned home.

At the 19-month post-ICU follow-up, the patient had resumed her previous daily routine. She remained wheelchair-dependent within her home and only went out when accompanied by her children. She continued to receive substantial home support, including nursing care, home aides, and meal delivery (IADL 4/8, CFS 7/9). Her only reported complaint was memory impairment, which was also noted by her children.


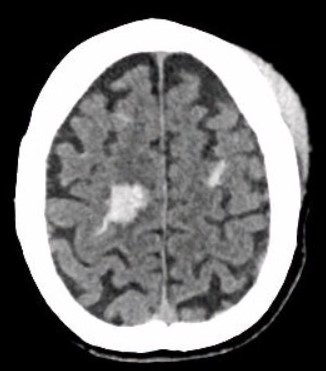

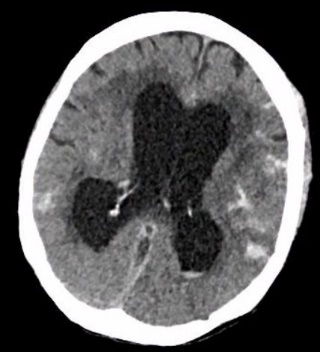

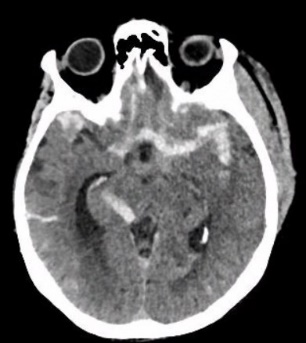


**Figure 8.** Brain CT scan of case #7 at ICU admission.

**Case #8**

A 72-year-old woman, married with two children and living independently at home (IADL 8/8, CFS 1/9), was admitted following a mild traumatic brain injury with an initial Glasgow Coma Scale (GCS) score of 13 (E4V4M5). The initial CT scan showed diffuse subarachnoid hemorrhage with a right frontal intraparenchymal contusion.

On day 3, she developed a focal motor status epilepticus affecting the right side, followed on day 4 by neurological and respiratory deterioration requiring mechanical ventilation. On day 6, she presented with refractory intracranial hypertension, leading to placement of an EVD.

She was extubated on day 26, and the EVD was removed on day 28. By day 36, her GCS was 14 (E4V4M6); she was able to verbalize simple words but presented with features of a frontal syndrome. She was subsequently transferred to the post-ICU rehabilitation unit.

At month 9, she was discharged from the post-ICU rehabilitation unit and transferred to a rehabilitation facility. She returned home at month 12.

At the 26-month post-ICU follow-up, the patient was living at home with her husband. She showed nearly complete recovery of fine motor skills but remained dyspneic with minimal exertion. Cognitive function was globally preserved, although she continued to experience word-finding difficulties. She received home support consisting of housekeeping assistance, caregiver help for personal hygiene, and weekday meal delivery (IADL 7/8, CFS 3/9).


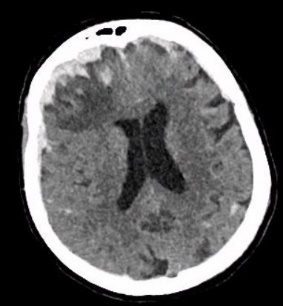

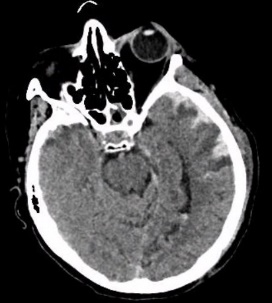


**Figure 9.** Brain CT scan of case #8 at ICU admission.

**Case #9**

A 75-year-old woman, married and living independently at home (IADL 8/8, CFS 2/9), was admitted following a severe traumatic brain injury with an initial GCS score of 7 (E1V1M5). The initial CT scan showed diffuse subarachnoid hemorrhage and a facial fracture.

She was admitted to the neurocritical care unit for 24 hours. Extubation was performed on day 1, and by day 2, she had recovered full consciousness with a GCS of 15, allowing transfer to the neurosurgery ward.

At month 2, she returned home.

At the 19-month post-ICU follow-up, the patient was living at home with her husband. She demonstrated excellent physical and cognitive recovery but reported significant anxiety that considerably limited her social interactions and ability to go out. She did not require any home assistance (IADL 6/8, CFS 2/9).


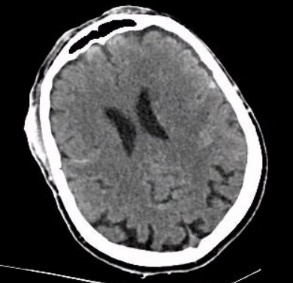

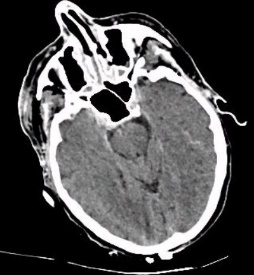


**Figure 10.** Brain CT scan of case #9 at ICU admission.
